# The natural history of ductal carcinoma in situ (DCIS) in simulation models: A systematic review

**DOI:** 10.1016/j.breast.2023.07.012

**Published:** 2023-07-27

**Authors:** Keris Poelhekken, Yixuan Lin, Marcel J.W. Greuter, Bert van der Vegt, Monique Dorrius, Geertruida H. de Bock

**Affiliations:** a University of Groningen, University Medical Center Groningen, Groningen, Department of Epidemiology, P.O. Box 30 001, FA40, 9700, RB, Groningen, the Netherlands; b University of Groningen, University Medical Center Groningen, Groningen, Department of Radiology, PO Box 30.001, EB44, 9700, RB, Groningen, the Netherlands; c University of Groningen, University Medical Center Groningen, Groningen, Department of Pathology and Medical Biology, PO Box 30.001, 9700, RB, Groningen, the Netherlands

**Keywords:** Breast neoplasms, Breast carcinoma in situ, Early detection of cancer, Computational modelling, Disease progression

## Abstract

**Objective:**

Assumptions on the natural history of ductal carcinoma in situ (DCIS) are necessary to accurately model it and estimate overdiagnosis. To improve current estimates of overdiagnosis (0–91%), the purpose of this review was to identify and analyse assumptions made in modelling studies on the natural history of DCIS in women.

Methods: A systematic review of English full-text articles using PubMed, Embase, and Web of Science was conducted up to February 6, 2023. Eligibility and all assessments were done independently by two reviewers. Risk of bias and quality assessments were performed. Discrepancies were resolved by consensus. Reader agreement was quantified with Cohen's kappa. Data extraction was performed with three forms on study characteristics, model assessment, and tumour progression.

**Results:**

Thirty models were distinguished. The most important assumptions regarding the natural history of DCIS were addition of non-progressive DCIS of 20–100%, classification of DCIS into three grades, where high grade DCIS had an increased chance of progression to invasive breast cancer (IBC), and regression possibilities of 1–4%, depending on age and grade. Other identified risk factors of progression of DCIS to IBC were younger age, birth cohort, larger tumour size, and individual risk.

**Conclusion:**

To accurately model the natural history of DCIS, aspects to consider are DCIS grades, non-progressive DCIS (9–80%), regression from DCIS to no cancer (below 10%), and use of well-established risk factors for progression probabilities (age). Improved knowledge on key factors to consider when studying DCIS can improve estimates of overdiagnosis and optimization of screening.

## Highlights

• 30 models on the natural history of ductal carcinoma in situ (DCIS) are identified.
• Non-progressive DCIS (8–90%) is important to consider in natural history models.
• <10% of DCIS regress and there is no regression of invasive breast cancer.
• Grades and age are well-established risk factors to include in DCIS models.
• Validation and uncertainty are underreported in modelling studies.

## 1 Introduction

Breast cancer has globally become the most diagnosed cancer type in 2020, with 2.3 million new breast cancer cases [1]. To detect breast cancer at an early stage, screening programs have been introduced in many countries. Before the implementation of screening, ductal carcinoma in situ (DCIS) was rarely diagnosed, where, nowadays, DCIS accounts for approximately 20–33% of all detected breast cancer cases [[2], [3], [4], [5]]. DCIS is considered as stage zero breast cancer and is defined as “a neoplastic proliferation of cells within the ductal-lobular structures of the breast that has not penetrated the myoepithelial-basement membrane interface” [2,3,6,7]. DCIS can be divided into low, intermediate, and high grade, where high grade is more likely to progress to invasive breast cancer (IBC) [5]. The proportion and grade distribution of DCIS detected in screening depends on the imaging modality used in the program. Although DCIS itself is not considered as life threatening and can remain indolent [3,7], the proportion of DCIS that will progress to IBC and the proportion that regresses if left untreated are unknown [8,9]. As a consequence, contradictory interpretations on this increased detection of DCIS arise in literature: whether it reflects the benefit of screening in detecting cancer at an early stage [6,8], or that it represents overdiagnosis [2,7,8].

Overdiagnosis, the proportion of breast cancers that would not have been diagnosed during a woman's lifetime in absence of screening, is considered to be one of the largest harms of screening [7]. Estimates of overdiagnosis vary largely (0%–91%) and depend on many factors, such as whether the estimate includes only IBC, only DCIS, or both [5,7,[9], [10], [11]]. Although there is consensus that DCIS is the largest contributor to overdiagnosis, the extent to which this occurs is unclear due to the unknown natural history of DCIS [3,6,10,12,13]. To make accurate estimates on overdiagnosis, and, as a result, to better weigh the benefits and harms of screening, more knowledge is needed on the natural history of DCIS [7,10]. To accurately estimate overdiagnosis a long follow-up time is necessary [7,9,10]. However, long follow-up times of screening in randomized controlled trials (RCTs) are very expensive [10]. Also, with DCIS considered as a risk factor for the development of invasive breast cancer, leaving DCIS untreated is considered unethical. Therefore, it is difficult to obtain accurate information on its natural history. Modelling studies provide a complementary method to make accurate estimates of tumour progression and overdiagnosis, with their ability to use long follow-up and account for both the benefits and harms of screening. Especially natural history models can provide insight into underlying processes and can be useful in assessment of the benefits and harms of screening scenarios [11]. However, modelling studies have shown a large range in assumptions that affect tumour progression and overdiagnosis estimates [2,3,11,13].

The aim of this study was, therefore, to identify and analyse assumptions made on the natural history of DCIS in modelling studies. Identifying assumptions on the natural history of DCIS can provide insight in how to simulate the development of DCIS and to better understand the development of IBC to estimate the extent of overdiagnosis in breast cancer screening more accurately.

## 2 Methods

### 2.1 Search strategy

A systematic literature search was conducted in PubMed, Embase, and Web of Science up to February 6, 2023. For this study, a PDO (population, determinant, outcome) was created, with women with DCIS as population, a model as determinant, and the progression of DCIS as outcome. Methods of the search strategy were specified in advance (PROSPERO ID CRD42022347862, Appendix A.1.).

### 2.2 Eligibility criteria

Included were full-text articles published in English, irrespective of the year of publication, which used a model to describe tumour progression of DCIS in all populations of women of all age groups. Of all modelling studies, the original model description, extensions, and applications of different versions of a specific model were included. If it was unclear during initial screening whether DCIS was included in a model and all other criteria were met, the study was included for full-text screening. Excluded were reviews and case reports, studies in males, animals, on a microscopic level, on the effect of specific treatments, and predictions of risk factors.

### 2.3 Study selection

Two authors (KP and YL/MG) independently reviewed potentially relevant studies for inclusion. Screening was performed first on title and abstract, and second on full text, based on the previously described eligibility criteria. Disagreements on eligibility were resolved by consensus. References of the selected publications were manually screened for other eligible studies. Reader agreement was quantified using Cohen's kappa for initial title/abstract screening, full-text screening, risk of bias assessment, and quality assessment [14].

### 2.4 Data extraction and analysis

Data extraction was done with three predefined forms: a form on general study characteristics, a form for model assessment, and a form for model characteristics on tumour progression. The latter was used to analyse the ways tumour progression of DCIS was modelled.

### 2.5 Study characteristics

The extracted and analysed general study characteristics included the model, publication year, type of study, population characteristics (years of input data, country, risk group), and screening characteristics (screening age, type, modality, and interval) (Appendix A.2.1.). The type of study was classified as original model, extension, description, or application. Original models were defined as the first description of the model. Extensions were defined as studies modifying or extending an original or extended model. Descriptions were defined as studies which described the model characteristics and its applications in detail, without adjustment or application of the model. Applications were defined as studies using an earlier described original or extended model without changing it. All studies were included in the first form. For each model, one study was selected as most informative and used to fully analyse the model and the progression of DCIS. This study was selected using the following criteria: an original or extension model of the general population with at least one natural history assumption mentioned. If multiple studies fulfilled these criteria, the most recent study was used.

### 2.6 Model assessment

The model assessment form included the model, a classification of model types, validation, sensitivity analysis (SA), risk of bias, and quality assessment (Appendix A.2.2.). The model type was assigned according to the classification of Brennan et al. [15] and was divided into eight categories (Appendix A.3.1.). The categories were based on the following characteristics: individual or cohort level simulation, (non-)Markovian, continuous or discrete time simulation, and the possibility of interaction [15]. Validation of the models was assessed based on data on face, internal, cross, and external validation [16]. Face validation was present if experts judged the model output as valid. Internal validation was present if the model output matched the data used for calibration of the model input. Cross validation was present if the model output was compared to output of other models. External validation was present if the model output was compared to data independent from the data used for calibration of the model input [16,17]. For judgement of the SA, two categories were considered: discrete and probabilistic. For discrete SA, only one input parameter was changed at a time and the effect on the model output was assessed. For probabilistic SA, multiple input parameters were changed at the same time by bootstrapping from a distribution.

The risk of bias was assessed according to the criteria for modelling studies developed by Carter et al. [10] (Appendix A.3.2.). Two reviewers (KP and YL) independently rated the risk of bias for each included study as low, moderate, or high risk of bias, and discrepancies were resolved by consensus. The quality of the included studies was assessed using the strength of evidence criteria developed by Carter et al. [10] adjusted to the current study *(*Appendix A.3.3.). Two reviewers (KP and YL) independently rated the quality of all included studies as high, moderate, or low. Conflicting decisions were resolved by consensus.

### 2.7 Tumour progression

The tumour progression form was made to collect all data on the natural history of DCIS. It included DCIS substages, progression dependencies, regression stages and dependencies, and natural history assumptions made (Appendix A.2.3.). The DCIS substages included all stages within the DCIS stage, such as progressive and non-progressive DCIS, and low-, intermediate-, and high-grade DCIS. The progression dependencies were defined as the characteristics influencing the progression of DCIS to a higher stage, such as age. For regression, the stage routing was collected as well as the characteristics the regression depended on. For all models, natural history assumptions were collected, for example whether DCIS was modelled as a precursor of invasive breast cancer.

## 3 Results

### 3.1 Search results

**Fig. 1:**
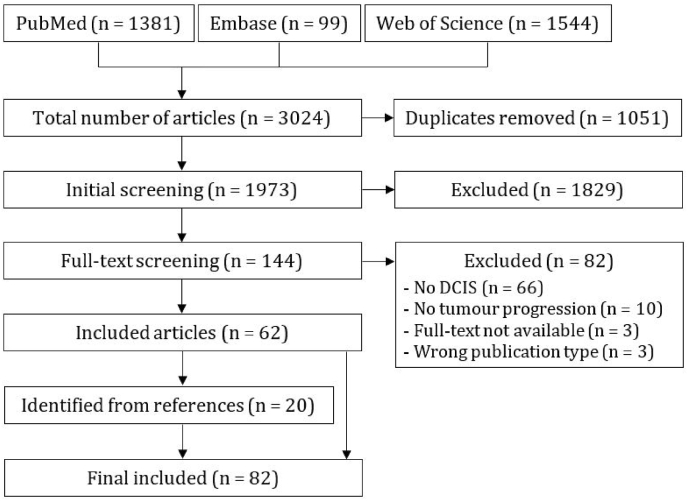
Flow diagram of identification of eligible studies.

The initial search of PubMed, Embase, and Web of Science resulted in 3024 articles for title and abstract screening, of which 62 were eligible after full-text screening (Fig. 1). From the references of these 62 studies, 20 additional studies were included. For both initial and full-text screening reader agreement was substantial (Appendix B.1.).

### 3.2 Study characteristics

For all 82 included studies, the general study characteristics were extracted and summarized (Appendix B.2.1.). We identified 34 different models, described in 30 original model studies, 12 extensions, 3 descriptions, and 37 applications. Four models did not make clear assumptions on the natural history of DCIS. There were 24 single-use models, and 10 models with multiple studies. Five multi-use models with 31 studies belonged to the Cancer Intervention and Surveillance Modelling Network (CISNET) Breast Cancer group [13,[18], [19], [20], [21]]. The MISCAN model was used in fifteen studies and is an earlier Dutch version of the MISCAN-FADIA model of the CISNET group [2]. The simulations focused on the United States (36), Europe (26), Asia (9), Canada (5), and South America (2). Besides the models simulating the general population, four models were race-specific [[22], [23], [24], [25]], four focused on high-risk groups [[26], [27], [28], [29]], and three on women with dense breasts [[30], [31], [32]]. The screening age varied, with a starting age of 30–50 and ending age 49–80, and the screening interval ranged from 6 months to 4 years. Next to population-based screening, three studies focused on opportunistic screening [[33], [34], [35]], and five studies included both [18,28,[36], [37], [38]]. The screening modalities included in the studies were screen-film mammography, digital mammography, ultrasound, magnetic resonance imaging, clinical breast examination, and tomosynthesis.

### 3.3 Model assessment

From the 34 identified models, 30 models made at least one assumption on tumour progression and were, therefore, fully assessed (Table 1) [2,8,13,[18], [19], [20], [21],^26^,^33^,^34^,^36^,[39], [40], [41], [42], [43], [44], [45], [46], [47], [48], [49], [50], [51], [52], [53], [54]]. We identified 12 cohort and 18 individual level models. Eight different model types were identified, of which the most frequently were simulated Markov models (SMM) (10) [8,[34], [35], [36], [37],^45^,^48^,^53^,^55^,^56^] and simulated patient-level Markov model (9) [2,13,18,20,42,43,46,51,52]. Only thirteen models reported external or multiple validation [[18], [19], [20], [21],^35^,^39^,^40^,^42^,^47^,^51^,^52^,^54^,^56^], and five models reported no validation [8,34,44,46,50]. Fourteen models did not address the uncertainty with a deterministic or probabilistic sensitivity analysis (SA) [2,8,13,18,19,21,33,36,39,41,42,[46], [47], [48]]. For the risk of bias, eight models were rated low (27%) [20,35,45,49,51,53,54,56], eighteen moderate (60%) [2,13,18,19,21,26,33,36,37,[39], [40], [41], [42], [43], [44],^47^,^50^,^52^], and four high (13%) [8,34,46,48] (Table 1). For the quality assessment, only two models were rated high (6%) [20,45], twenty moderate (67%) [2,13,18,19,21,26,33,36,[39], [40], [41], [42],^47^,[49], [50], [51], [52], [53],^56^,^57^], and eight low quality (27%) [8,34,35,37,43,44,46,48] (Table 1). Reader agreement for the risk of bias and quality assessment was substantial (Appendix B.1.).

**Table 1:** Model assessment.

Model [reference]	Model type^a^	Validation^b^	Sensitivity Analysis^c^	Risk of bias^d^	Quality^e^
Carter [39]	CT, DES	E	None	Moderate	Moderate
CISNET D [13]	SPLMM	I	None	Moderate	Moderate
CISNET E [18]	SPLMM	F,I,C,E	None	Moderate	Moderate
CISNET GE [19]	CT, IEH	E	None	Moderate	Moderate
CISNET M [20]	SPLMM	E, C	D	Low	High
CISNET W [21]	DT, DES	I, C, E	None	Moderate	Moderate
Comas [40]	DT, DES	I,E	P	Moderate	Moderate
DES SD [41]	DT, DES	I	None	Moderate	Moderate
Forastero [42]	SPLMM	E	None	Moderate	Moderate
Gocgun [43]	SPLMM	I	D	Moderate	Low
Gray [44]	CT, DES	None	D, P	Moderate	Low
Gunsoy [45]	SMM	I	P	Low	High
Huang [33]	DTMC	I	None	Moderate	Moderate
Hunter [46]	SPLMM	None	None	High	Low
MISCAN [2]	SPLMM	I	None	Moderate	Moderate
OncoSim-Breast [47]	CT, DES	F, C, E	None	Moderate	Moderate
Ozanne [48]	SMM	I	None	High	Low
POMDP [26]	DT, IEH	C	D	Moderate	Moderate
Rafia [49]	CT, IEH	I	D, P	Low	Moderate
Rojnik [35]	SMM	E	D, P	Low	Low
Ryser [50]	DTMC	None	D, P	Moderate	Moderate
Schiller-Fruehwirth [51]	SPLMM	I, C	D, P	Low	Moderate
Schousboe [56]	SMM	I, E	D, P	Low	Moderate
Seigneurin [37]	SMM	I	D	Moderate	Low
Souza [52]	SPLMM	E	D, P	Moderate	Moderate
Tan [53]	SMM	I	D	Low	Moderate
Weedon-Fekjaer [36]	SMM	I	None	Moderate	Moderate
Wong [54]	SMM	E	P	Low	Moderate
Yang [34]	SMM	None	D, P	High	Low
Yen [8]	SMM	None	None	High	Low

a Model type (Appendix A.3.1): discrete event simulation (CT, DES), simulated patient-level Markov model (SPLMM), continuous/discrete time individual event history model (CT/DT, IEH), discrete individual simulation (DT, DES), simulated Markov model (SMM), discrete time Markov chain model (DTMC).

b Type of validation: face (F), internal (I), cross (C), external (E), or not reported (None).

c Sensitivity analysis performed: deterministic (D), probabilistic (P), or not reported (None).

d Risk of bias assessed with Appendix A.3.2.

e Quality assessed with Appendix A.3.3.

### 3.4 DCIS progression

**Fig. 2:**
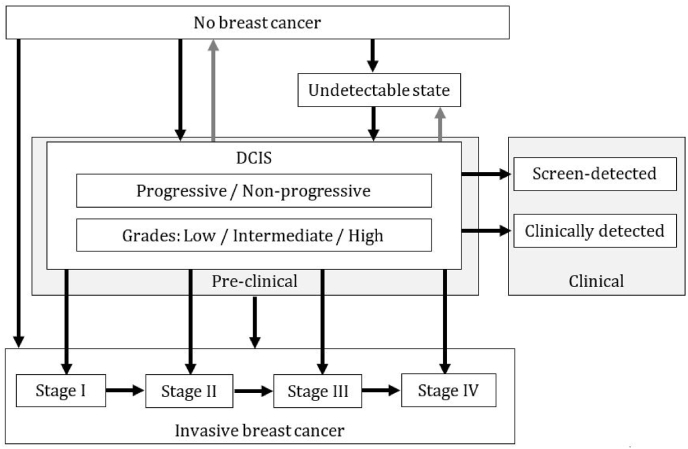
Schematic overview of the natural history of DCIS transitions found in the models. Arrows indicate a pathway for progression (black), or regression (gray). From a state of no breast cancer progression can occur either to DCIS, to an undetectable state, or directly to IBC. DCIS can progress to a general state of IBC or to a specific stage. Models included a non-progressive fraction of DCIS or grades of DCIS. Models often separated pre-clinical stages, before detection, and clinical stages, after screen- or clinical detection. Regression was found only from DCIS stage to no breast cancer or an undetectable state.

The models used various natural history pathways to simulate DCIS (Fig. 2). From a no breast cancer stage, progression was possible to a DCIS stage in all models (Table 2). The DCIS stage was either a general DCIS state (14) or a substage. The substages included were non-progressive and progressive (7), pre-clinical and clinical (6), undetectable and detectable (2), grades of DCIS (2), or a combination of these substages. The grades of DCIS described the progression potential of DCIS, with high-grade having a higher probability of progression and a lower possibility of regression [2]. Models including grading, reported 18–30% low, 30–31% intermediate, and 40–51% high grade DCIS, with a progression risk of 15–16%, 21–31%, and 30–60%, and regression of 4%, 2%, and 1% respectively. Progression from no breast cancer to DCIS and DCIS to IBC was dependent on age (21), birth cohort (4), tumour size (3), or individualised risk factors (1). In eleven models, regression was possible from a DCIS stage to either no breast cancer or to an undetectable state. Only three studies explicitly reported the percentage of DCIS that could regress, ranging from 1 to 4%. The CISNET W model included a low malignant potential (LMP) fraction of 42% of total DCIS, that could regress [21]. No model included regression from IBC to DCIS or no breast cancer (Fig. 2).

**Table 2:** Characteristics DCIS.

Model [reference]	Substages^a^	Progression	Regression^b^	
	In situ	depends on	stage	depends on (% regression)
Carter [39]	DCIS	–	–	–
CISNET D [13]	UIS, DIS, CIS	Age	DIS > UIS	–
CISNET E [18]	UIS, DIS, CIS	Age, birth cohort	DIS > UIS	Age (1%–3%)
CISNET GE [19]	NPIS, PIS	Age	NPIS > No bc	–
CISNET M [20]	DCIS	Age	–	–
CISNET W [21]	DCIS (LMP)	Age, birth cohort	DCIS > No bc	LMP (<42%)
Comas [40]	CIS, PCIS	Age, birth cohort	–	–
DES SD [41]	DCIS	–	–	–
Forastero [42]	LCIS, DCIS	Tumour size	–	–
Gocgun [43]	DCIS	Age	–	–
Gray [44]	DCIS	Age	–	–
Gunsoy [45]	NPIS, PIS, SDIS	Age	–	–
Huang [33]	CIS, PCIS	Age	–	–
Hunter [46]	DCIS	Age	–	–
MISCAN [2]	Grades, CIS, PCIS	Age	DCIS > No bc	Grade (1–4%)
OncoSim-Breast [47]	NPIS, PIS	Age, birth cohort	–	–
Ozanne [48]	Grades	Age, tumour size	–	–
POMDP [26]	DCIS	Age, individual risk	–	–
Rafia [49]	DCIS	Age	–	–
Rojnik [35]	DCIS	Age	DCIS > No bc	–
Ryser [50]	NPIS, PIS	Age	DCIS > No bc	–
Schiller-Fruehwirth [51]	CIS, PCIS, SDIS	–	DCIS > No bc	Time (1.75%)
Schousboe [56]	DCIS	–	–	–
Seigneurin [37]	NPIS, PIS, CIS	Age	–	–
Souza [52]	DCIS	–	DCIS > No bc	–
Tan [53]	NPIS, PIS	Tumour size	–	–
Weedon-Fekjaer [36]	CIS, PCIS, NPIS	–	DCIS > UIS	–
Wong [54]	DCIS	Age	–	–
Yang [34]	DCIS	Age	–	–
Yen [8]	NPIS, PIS	Age	DCIS > No bc	program

a Substages: ductal carcinoma in situ as one general stage (DCIS), non-progressive in situ (NPIS), progressive in situ (PIS), clinical in situ (CIS), pre-clinical in situ (PCIS), undetectable in situ (UIS), detectable in situ (DIS), lobular carcinoma in situ (LCIS), screen-detected in situ (SDIS), low malignant potential (LMP), ductal carcinoma in situ subdivided in grades (Grades).

b Regression: no regression (−), to no breast cancer (No bc), from stage to stage (>). Between brackets: percentage of DCIS that can regress.

A total of 25 models simulated DCIS as a precursor of IBC, where five models used DCIS as a separate stage of breast cancer (Table 3). Progression of DCIS to IBC was possible to a general invasive stage (12), only to stage I IBC (9), or to all stages (4). Only ten models had a mandatory DCIS stage, where direct progression from no breast cancer to IBC was not possible. A fraction of non-progressive DCIS was included in fifteen models, ranging from 20 to 100%. In only five models, DCIS led to an increased possibility of death compared to death from other causes.

**Table 3:** Assumptions DCIS.

Model	Precursor of IBC^a^	Progression IBC stages^b^	Mandatory DCIS state^c^	Non-progressive DCIS^d^	Lethal DCIS^e^
Carter [39]	Yes	I	ns	–	–
CISNET D [13]	Yes	General	Yes	Yes	–
CISNET E [18]	Yes	General	Yes	–	–
CISNET GE [19]	Yes (80%)	General	ns	Yes (20%)	80%
CISNET M [20]	–	–	–	–	–
CISNET W [21]	Yes	General	Yes	–	Yes
Comas [40]	–	–	–	–	ns
DES SD [41]	–	–	–	Yes (100%)	–
Forastero [42]	Yes (0–80%)	General	–	≤ 6 mm	ns
Gocgun [43]	Yes	I	Yes	–	–
Gray [44]	–	–	–	Yes (100%)	–
Gunsoy [45]	Yes	General	Yes	Yes	Yes
Huang [33]	Yes	I	–	–	–
Hunter [46]	Yes (25.9/29.9%)	I	Yes	–	Yes
MISCAN [2]	Yes (16/31/53%)	I	Yes	–	–
OncoSim-Breast [47]	Yes	General	–	–	–
Ozanne [48]	Yes (0–100%)	General	–	Yes (0–100%)	ns
POMDP [26]	Yes	General	–	–	–
Rafia [49]	–	–	–	–	ns
Rojnik [35]	Yes (65%)	I	- (40%)	Yes (35%)	–
Ryser [50]	Yes (10–70%)	I	ns	Yes	ns
Schiller-Fruehwirth [51]	Yes (65%)	I	–	Yes (35%)	Yes
Schousboe [56]	Yes	I, II, III, IV	–	Yes	–
Seigneurin [37]	Yes	General	–	Yes	–
Souza [52]	Yes	I, IV	–	–	0.2%/y
Tan [53]	Yes	I	Yes	Yes	ns
Weedon-Fekjaer [36]	Yes	General	Yes	Yes	–
Wong [54]	Yes	I,II,III,IV	–	–	–
Yang [34]	Yes	I,II,III,IV	–	–	–
Yen [8]	Yes	General	Yes	Yes (37%)	ns

a DCIS as precursor of invasive breast cancer (IBC): Yes/No (−). Between brackets: percentage precursor of IBC.

b IBC stages DCIS can progress to: stage I (I)/stage II (II)/stage III (III)/stage IV (IV), no specific stage (General).

c Mandatory DCIS state: Yes/No (−), Not specified (ns). Between brackets: percentage IBC with mandatory DCIS precursor.

d Mandatory DCIS state: Yes/No (−). Between brackets: percentage non-progressive DCIS.

e Lethal DCIS: Yes/No (−)/Fraction (%)/Not specified (ns).

## 4 Discussion

The aim of this study was to identify and analyse the assumptions made to model the natural history of DCIS. Thirty models that simulate DCIS and made at least one assumption on its natural history were identified and fully assessed. The most prominent assumptions regarding the natural history of DCIS were addition of non-progressive DCIS, grading, progression dependencies, and regression possibilities. DCIS was modelled as a general state able to progress to IBC or separated into a progressive and non-progressive fraction of 20–100%. DCIS was divided into three grades in two models, where a high DCIS grade increased the chance of progression to IBC. Other identified risk factors of progression of DCIS to IBC were younger age, birth cohort, larger DCIS size, and individual risk. The fraction of DCIS able to regress was 1–4% and depended on various factors, such as age and grade. Most models (30%) were individual-level state-transition models. Model validation and uncertainty were not addressed in 17% and 47% of the models, respectively, and risk of bias and quality were assessed as moderate in 60% and 67% of the models, respectively.

Assumptions on DCIS progression are necessary to accurately model the natural history of DCIS. In recent years, it has become clear that a fraction of DCIS detected by mammography progresses very slowly, or is even indolent [5,58]. Although the exact fraction of indolent DCIS is unknown, it is these non-progressive and slow growing DCIS fractions that are most likely to add to overdiagnosis. Models simulating the natural history of DCIS used a non-progressive fraction of 20–100%. Some studies did not simulate DCIS as a precursor of IBC, which is reflected in models by a 100% non-progressive DCIS fraction. However, DCIS as a completely separate state from IBC is deemed unlikely due to overlapping genetic profiles and transitional states from in situ to IBC being noted in histopathology [5,59]. The size of the indolent fraction also depends on other model assumptions, such as whether all IBC has a DCIS precursor or not, since some tumours appear to have skipped the DCIS state and directly became invasive [60]. Exact estimates of non-progressive DCIS are not possible to obtain, since leaving DCIS untreated is considered unethical. Based on previous literature, it is recommended to implement a non-progressive DCIS fraction of 9–80% in the model, but the exact size of this fraction remains unclear [60].

To model the natural history of DCIS, DCIS should be divided into three grades (low, intermediate, and high), with their own risk of progression [5], as was done in the identified models [2]. A large study by Van Luijt et al. [2] showed a distribution of 18–30%, 30–31%, and 40–51% low-, intermediate-, and high-grade DCIS, respectively, providing valuable input to model the natural history of DCIS. Previous pathology studies showed similar estimates of 14% low-, 43% intermediate-, and 42% high-grade [5]. However, variations in distributions are common, since there is a large inter-reader variation in grading, especially because of difficulties to distinguish the intermediate-grade [61]. Furthermore, pathology studies have shown an increased progression probability for higher grade DCIS [5]. Models that included grading reported a risk of progression of 15–16%, 21–31%, and 30–60% for low-, intermediate-, and high-grade, respectively. Unfortunately, the exact distribution and progression risk per grade are not known and unobservable, but the estimates are comparable. Therefore, new biological information and modelling estimates are crucial to improve these estimates. Moreover, there is no progression between DCIS grades [2]. Up-to-date information on the distribution of grades, the progression risk, and progression pathways should therefore be used to model DCIS natural history.

Besides DCIS grade, other factors associated with increased risk of DCIS onset and progression to IBC were identified in the models. The most important factor to consider in risk of DCIS onset and progression is age, with a higher chance being diagnosed with DCIS at older age, and young age associated with increased risk of progression to IBC [62]. Furthermore, the risk factor birth cohort is only necessary to implement in a model when the incidence is calibrated to specific years, and the effect of age and introduction of screening is combined. In addition, larger DCIS size had increased risk of progression in three models, while tumour size was only associated with recurrence risk of DCIS in previous studies, and not with risk of onset or progression [62]. To model the natural history of DCIS, it is recommended to only implement well-established risk factors, such as age.

Regression of DCIS should also be considered when modelling the natural history to get accurate estimates of overdiagnosis [58,63]. Studies suggest a maximum 10% total regression rate of breast cancer, although evidence is limited [58]. In the identified models, regression of DCIS ranged from 1 to 4%, but was often not clearly specified, and different factors determined the chance of regression. Only the model of Van Luijt et al. [2] varied probability of regression per grade, with a decreased probability of regression at higher grades, while other studies showed that regression was more prevalent in higher grade DCIS [63]. No model included regression from IBC to DCIS, which was in line with literature reporting this as rare [63]. More accurate information on regression, but also on grade distribution and progression, is expected to come from ongoing trials that implemented a watchful waiting approach for low-grade DCIS [5].

Important to consider in modelling the natural history of DCIS is the screening program design, because a change in screening modality and screening interval influences the observed chance of detection, and, therefore, the observed risk of progression [62]. The screening modalities differ in their capabilities to detect high-grade DCIS. Therefore, the used modality also partly determines the observed grade distribution, the risk of progression of detected versus undetected DCIS, and of the risk of IBC. It is recommended to use a progression risk and grade distribution of DCIS detected in screening that matches the screening program design.

To model the natural history of DCIS variations in individual-level, cohort, state-transition, and event simulation models were identified. An individual-level state-transition model improves validity of more complicated disease models, and is more flexible, but more iterations are needed to attain a stable result [17,64]. Therefore, cohort models are recommended when the disease and outcome allow for more simplicity [64], which was also visible in the identified models. Although clear guidelines exist for modelling studies regarding what to report, and how to validate and address uncertainty [64], these recommendations were often not accurately followed [17], with five and fourteen models reporting no validation and uncertainty, respectively. Furthermore, use of high-quality input data is crucial to obtain high quality outcomes with low risk of bias. The quality of the input data was assessed in the risk of bias (category ‘Bias Data’) and quality assessment (category ‘External Validity’), which showed only 16 models with low risk of bias in the input data and 15 models with high quality input data (Appendix B.3). This highlights the importance of assessing the input data in risk of bias and quality assessment. The risk of bias and quality were assessed as moderate for 60% and 67% of the models, respectively. Given the unknown natural history of DCIS and especially the necessity to use unobservable data as input, it is crucial that uncertainty and validation are properly addressed and the input data is assessed.

This systematic review has some limitations that could be considered. First, the aim of several models was to estimate cost-effectiveness of screening or estimate outcomes on overall breast cancer, instead of focusing on DCIS specific outcomes. Therefore, the natural history of DCIS was not reflected in detail in these models. However, these models also highlight the possibility of simplifications in the natural history when interested in specific outcomes. Second, assumptions were analysed based on a general population, but it is deemed unlikely that there is one perfect model for all populations. To model the natural history of DCIS, population characteristics should be considered. Third, in the manual search 25% additional articles were identified, showing the difficulty to construct an ultimate search strategy on DCIS. However, all but three were applications of already identified models, which gave confidence that most DCIS models were identified in the original screening. Fourth, only English articles were included. However, this is not expected to have a substantial impact since over 95% of the originally identified studies were English. Fifth, treatment models were excluded from this study and considered outside of the scope. As a result, models including lack of endocrine therapy as a risk factor could have been excluded, although this is a known risk factor for progression of DCIS to IBC [62].

Also, several strengths should be considered. This review focused on the identification of all natural history models on DCIS. To make sure most models were identified the eligibility criteria were kept broad, to identify all article types, study settings, and model types. Furthermore, it was often not specified in the title or abstract whether DCIS was included in the model or only briefly mentioned, so when in doubt these articles were screened full text. In addition, inter-reader agreement was substantial to near perfect, which indicates reliable selection and assessment of the studies (Appendix B.1.). Although DCIS only accounts for 20% of breast cancer cases, it is considered as the largest contributor to overdiagnosis and large gaps in knowledge exist [5,11,62]. This review can contribute to a better understanding of the key factors which have to be considered when studying DCIS, and therefore, in potentially better estimates of overdiagnosis and optimization of screening.

## 5 Conclusion

In conclusion, to accurately model the natural history of DCIS it is recommended to include a non-progressive fraction of 9–80% and DCIS grades, where progression depends on well-established risk factors such as high-grade and age, and to exclude progression between grades and regression from IBC to DCIS. Whether regression from DCIS to no breast cancer must be included depends on the purpose of the model but should be less than 10%. In addition, validation and uncertainty should be addressed in modelling studies. Future results from ongoing active surveillance trials may lead to an increased knowledge on the natural history of DCIS, with the possibility to improve current overdiagnosis estimates of 0–91%.

## Funding

KPis supported by the W.J. Kolff Institute (WJKI) for her PhD study. The WJKI had no involvement in the study.

## Ethical approval

Ethical approval was not required.

## CRediT authorship contribution statement

**Keris Poelhekken:** Conceptualization, Methodology, Software, Validation, Formal analysis, Investigation, Resources, Data curation, Writing – original draft, Writing – review & editing, Visualization, Project administration. **Yixuan Lin:** Methodology, Software, Formal analysis, Investigation, Writing – original draft, Writing – review & editing. **Marcel J.W. Greuter:** Conceptualization, Methodology, Software, Validation, Formal analysis, Investigation, Resources, Writing – original draft, Writing – review & editing, Visualization, Supervision, Project administration. **Bert van der Vegt:** Formal analysis, Resources, Writing – review & editing, Visualization. **Monique Dorrius:** Formal analysis, Resources, Writing – review & editing, Visualization. **Geertruida H. de Bock:** Conceptualization, Methodology, Software, Validation, Formal analysis, Investigation, Resources, Writing – original draft, Writing – review & editing, Visualization, Supervision, Project administration.

## Declaration of competing interest

The authors declare the following financial interests/personal relationships which may be considered as potential competing interests:BvdV declares advisory board/consultancy (on request) for Visiopharm, 10.13039/100004320Philips, MSD /10.13039/100004334Merck, 10.13039/501100002973Daiichi-Sankyo/AstraZenica and speaker's fee from Visiopharm, Diaceutics, MSD /10.13039/100004334Merck. All honoraria to UMCG. All unrelated to the current manuscript.
