# Supplementary material for: The natural history of ductal carcinoma in situ (DCIS) in simulation models: A systematic review
Source: Breast. 2023 Jul 27;71:74–81. doi: 10.1016/j.breast.2023.07.012 (PMC10412870; doi:10.1016/j.breast.2023.07.012)
Supplement: Multimedia component 1 [file mmc1.docx]

# Appendix

1. Methods
   1. Search strategy
      1. PDO and eligibility criteria
      2. Search strings
   2. Data extraction forms
      1. Study characteristics form
      2. Model assessment form
      3. Tumour progression form
   3. Assessment criteria
      1. Criteria model type assessment
      2. Criteria risk of bias assessment
      3. Criteria quality assessment
2. Results
   1. Reader agreement – Cohen’s kappa
      1. Initial screening
      2. Full-text screening
      3. Risk of bias assessment
      4. Quality assessment
   2. Additional Tables
      1. Model overview - General characteristics
         1. Original, extensions, and descriptions
         2. Applications
   3. Assessment results
      1. Risk of bias
      2. Quality

# Appendix A - Methods

## Appendix A.1. Search Strategy

## Appendix A.1.1. PDO and Eligibility criteria

|  | Include | Exclude |
| --- | --- | --- |
| Population | Women with ductal carcinoma in situ (DCIS) of all age groups from all populations | No breast cancer or DCIS included  Men, animals, in vivo, in vitro (microscopic level) |
| Determinant | Model | Models on specific treatments  Models to predict risk factors |
| Outcome | Tumour progression and regression  - Substages used  - Transition probabilities  - Assumptions | Models without tumour progression  Models without stages |
| Publication | Any publication date  Published language: English  Full-text articles |  |
| Setting | With a population-based screening |  |
| Study Design | Modelling studies | Non-modelling studies, case reports, reviews |

## Appendix A.1.2. Search Strings

| Database | Search String |
| --- | --- |
| PubMed | ("Breast Neoplasms"[Mesh] OR breast cancer*[tiab] OR “breast tumo*”[tiab] OR “breast carcinoma*”[tiab])  AND  ("Early Detection of Cancer"[Mesh] OR "Mammography"[Mesh] OR "Mass Screening"[Mesh:NoExp] OR screen*[tiab] OR mammogra*[tiab] OR early detect*[tiab]) AND  ("Computer Simulation"[Mesh:NoExp] OR "Models, Statistical"[Mesh:NoExp] OR "Markov Chains"[Mesh] OR model*[tiab] OR simulat*[tiab] OR markov[tiab])  AND  ("Disease Progression"[Mesh] OR progression[tiab] OR ‘natural history’[tiab] OR transition[tiab] OR growth[tiab])  NOT  (("Animals"[Mesh] NOT "Humans"[Mesh]) OR "Case Reports" [Publication Type] OR "Review" [Publication Type]) |
| Embase | ('breast cancer'/exp OR 'breast carcinoma'/exp OR 'breast tumor'/exp)  AND  ('early cancer diagnosis'/exp OR 'mammography'/exp OR 'mass screening'/exp OR screening:ti,ab,kw)  AND  ('computer simulation'/exp OR 'statistical model'/exp OR model:ti,ab,kw OR 'markov chain'/exp OR simulation:ti,ab,kw)  AND  ('disease exacerbation'/exp OR progression:ti,ab,kw OR 'natural history':ti,ab,kw OR growth:ti,ab,kw OR transition:ti,ab,kw)  NOT 'animal'/exp NOT review:it NOT 'case report':it |
| Web of Science | (TI=(breast neoplasm*) OR TI=(breast cancer*) OR TI=(breast tumo*) OR TI=(breast carcinoma*) OR AB=(breast neoplasm*) OR AB=(breast cancer*) OR AB=(breast tumo*) OR AB=(breast carcinoma*))  AND  (TI=(screen*) OR TI=(mammogra*) OR TI=(early detect*) OR AB=(screen*) OR AB=(mammogra*) OR AB=(early detect*))  AND  (TI=(model*) OR TI=(simulat*) OR TI=(markov) OR AB=(model*) OR AB=(simulat*) OR AB=(markov))  AND  (TI=(progression) OR TI=(natural history) OR TI=(transition) OR TI=(growth) OR AB=(progression) OR AB=(natural history) OR AB=(transition) OR AB=(growth))  NOT DT=(Review) NOT TI=(animal*) NOT AB=(animal*) |

## Appendix A.2. Data extraction forms

## Appendix A.2.1. Study characteristics form

| Model | Publication year | Study type | Years of input data | Country simulated | Population  risk group | Screening age | Screening type | Screening modality | Screening  interval |
| --- | --- | --- | --- | --- | --- | --- | --- | --- | --- |
| Model name  / First author | Year | O / E / D / A | Year range | Country simulated shortened | General  / High risk  / Specific population | Age range  / Varying | P / O | FM / DM  / US / MRI  / CBE | Annual  / Biennial  / Triennial  / Varying |

**For all categories**: - = not specified.

**Study type**: O = original model description, E = extension of an existing model, D = description of a model, A = application of a model.

**Screening type**: P = population-based screening, O = opportunistic screening.

**Screening modality**: FM = film screen mammography, DM = digital mammography, US = ultrasound, MRI = magnetic resonance imaging, CBE = clinical breast examination.

## Appendix A.2.2. Model assessment form

| Model | Model type | | | | Risk of bias | | | | | Quality | | | | |
| --- | --- | --- | --- | --- | --- | --- | --- | --- | --- | --- | --- | --- | --- | --- |
|  | Level | Markov | Time | Interaction | Transparency | Evidence | Bias data | Uncertainty | Validation | Risk of bias | Directness | External Validity | Precision | Consistency |
| Model name  / First author | Individual  / Cohort | Yes  / No | Continuous / Discrete | Yes  / No | Good  / Fair  / Poor | Good  / Fair  / Poor | Good  / Fair  / Poor | None  / P  / D | None  / F  / I  / C  / E | Low  / Mode-  rate  / High | Good  / Fair  / Poor | Good  / Fair  / Poor | Good  / Fair  / Poor | Good  / Fair  / Poor |

**Model type**: according to criteria Brennan et al. (Appendix A.3.1.).

**Risk of bias**: according to criteria Carter et al. (Appendix A.3.2.).

**Quality**: according to criteria Carter et al. (Appendix A.3.3.).

**Uncertainty**: None = no sensitivity analysis (SA), P = probabilistic SA, D = deterministic SA.

**Validation**: None = no validation done, F = face validation, I = internal validation, C = cross validation, E = external validation.

## Appendix A.2.3. Tumour progression form

| Model | Substages | Progression | | | Regression | | | | Assumptions |
| --- | --- | --- | --- | --- | --- | --- | --- | --- | --- |
|  | DCIS | Probability | Fractions | Depends on | Possible | Stages | Probability | Depends on | Natural history assumptions |
| Model name  / First author | DCIS / Grades  / NPIS / PIS  / CIS / PCIS  / UIS / DIS  / LCIS / SDIS  / LMP | % progression | Important fractions of progression or non-progression | Traits progression depends on | Yes / No | Stages regression possible | % regression | Traits progression depends on | All assumptions made, such as:  - DCIS a precursor of invasive? - To which stages can DCIS progress?  - Mandatory DCIS state?  - Other important fractions?  - DCIS survival percentage? |

**Substages**: ductal carcinoma in situ as one general stage (DCIS), ductal carcinoma in situ subdivided in grades (Grades), non-progressive in situ (NPIS), progressive in situ (PIS), clinical in situ (CIS), pre-clinical in situ (PCIS), undetectable in situ (UIS), detectable in situ (DIS), lobular carcinoma in situ (LCIS), screen-detected in situ (SDIS), low malignant potential (LMP).

## Appendix A.3. Assessment criteria

## Appendix A.3.1. Criteria model type assessment

| Model type | | Level | Markov | Time | Interaction |
| --- | --- | --- | --- | --- | --- |
| Discrete event simulation | CT, DES | Individual | No | Continuous | Yes |
| Discrete individual simulation | DT, DES | Individual | No | Discrete | Yes |
| Discrete time individual event history | DT, IEH | Individual | Yes | Discrete | Yes |
| Continuous time individual event history | CT, IEH | Individual | Yes | Continuous | Yes |
| Simulated patient-level Markov | SPLMM | Individual | Yes | Continuous/  Discrete | No |
| Simulated Markov | SMM | Cohort | Yes | Continuous/  Discrete | No |
| Discrete time Markov chain | DTMC | Cohort | Yes | Discrete | Yes |
| Continuous time Markov chain | CTMC | Cohort | Yes | Continuous | Yes |

## Appendix A.3.2. Criteria risk of bias assessment

| Criteria | Rating | Explanation |
| --- | --- | --- |
| Transparency | *Assumptions made in model are transparent*[1] | |
|  | Good | Nontechnical and technical aspects described |
|  | Fair | A few missing aspects |
|  | Poor | Not stated/many missing aspects |
| Evidence | *Assumptions made in model are backed up with evidence* | |
|  | Good | Systematically reviewed evidence, critically appraised with quality ratings, or almost no assumptions made |
|  | Fair | Solid evidence given |
|  | Poor | No evidence given/evidence missing |
| Bias Data | *Probability for bias in the data used in model*  *Measurement of outcomes and measurement of and control for potential cofounders* | |
|  | Good | Valid and reliable measurement adequate measurement |
|  | Fair | Measurements/information missing |
|  | Poor | Limited or no measurements/information given |
|  | Cannot determine | Probability for bias in data cannot be determined due to missing information |
| Uncertainty | *Sensitivity analysis (SA) performed to determine uncertainties variables and outcomes* | |
|  | Good | Probabilistic / multivariate SA or similar, and results reported |
|  | Fair | Deterministic / univariate SA or similar |
|  | Poor | No SA, or no results reported |
| Validation | *Model was validated using data* | |
|  | Good | The model was fully validated (external validation or multiple validations) |
|  | Fair | A validation was performed (internal or cross validation) and reported |
|  | Poor | No validation, or no results reported |

## Appendix A.3.3. Criteria quality assessment

| Criteria | Rating | Explanation |
| --- | --- | --- |
| Risk of bias | *Following criteria from Appendix Table 3* | |
|  | Low | Meets all criteria |
|  | Moderate | Do not meet all criteria, but no fatal flaw |
|  | High | Fatal flaw making results uncertain |
| Directness | *Screening directly linked to health outcomes by evidence with minimal assumptions regarding progression and association natural history with cancer progression and cancer-related morbidity and mortality* | |
|  | Good | Minimal assumptions, mostly directly from evidence |
|  | Fair | Some assumptions, backed by evidence |
|  | Poor | Many assumptions, not backed by evidence |
| External Validity | *Simulated population similar to true population*  *Population, screening situation, medical care, and risks for competing mortality* | |
|  | Good | All aspects similar to true population |
|  | Fair | Most aspects populations similar and no fatal flaw |
|  | Poor | Populations deviate completely or fatal flaw |
| Precision | *Precision of data is quantified* | |
|  | Good | Confidence interval given and narrow (<20%) |
|  | Fair | Confidence interval given but broad, not given everywhere, or other quantification used |
|  | Poor | Not quantified |
|  | Cannot determine | Missing information |
| Consistency | *Consistency of measurements between included studies* | |
|  | Good | Outcomes similar to studies with similar study design |
|  | Fair | Some deviations in outcomes |
|  | Poor | Outcomes deviate largely |

# Appendix B - Results

## Appendix B.1. Reader agreement - Cohen’s kappa

## Appendix B.1.1. – Initial screening

| Reader | | YL | | |
| --- | --- | --- | --- | --- |
|  |  | Include | Exclude | Total |
| KP | Include | 99 | 43 | 142 |
|  | Exclude | 34 | 1797 | 1831 |
|  | Total | 133 | 1840 | 1973 |
|  |  | **Kappa (95%CI)** | **0.699 (0.636-0.763)** | |

## Appendix B.1.2. – Full-text screening

| Reader | | YL | | |
| --- | --- | --- | --- | --- |
|  |  | Include | Exclude | Total |
| KP | Include | 54 | 8 | 62 |
|  | Exclude | 8 | 74 | 82 |
|  | Total | 62 | 82 | 144 |
|  |  | **Kappa (95%CI)** | **0.773 (0.669-0.878)** | |

## Appendix B.1.3. – Risk of bias assessment

| Reader | | YL | | | | |
| --- | --- | --- | --- | --- | --- | --- |
|  |  | Low | Moderate | | High | Total |
| KP | Low | 58 | 7 | | 0 | 65 |
|  | Moderate | 9 | 57 | | 0 | 66 |
|  | High | 0 | 1 | | 18 | 19 |
|  | Total | 67 | 65 | | 18 | 150 |
|  |  | **Kappa (95%CI)** | | **0.811 (0.725 - 0.897)** | | |

## Appendix B.1.4. – Quality assessment

| Reader | | YL | | | | |
| --- | --- | --- | --- | --- | --- | --- |
|  |  | High | Moderate | | Low | Total |
| KP | High | 38 | 6 | | 1 | 45 |
|  | Moderate | 16 | 62 | | 8 | 86 |
|  | Low | 1 | 1 | | 17 | 19 |
|  | Total | 55 | 69 | | 26 | 150 |
|  |  | **Kappa (95%CI)** | | **0.636 (0.527 - 0.745)** | | |

## Appendix B.2. Additional Tables

## Appendix B.2.1. – Model overview – General characteristics – A) Original, extension, and description

| Model | Publication year | Study type^a^ | Years of input data | Area simulated^b^ | Population  risk group^c^ | Screening age | Screening type^d^ | Screening modality^e^ | Screening  interval | Reference |
| --- | --- | --- | --- | --- | --- | --- | --- | --- | --- | --- |
| Carter | 2003 | O | 1983-1987 | USA | General | Varying | P | FM | Varying | [2] |
| CISNET D | 2018 | E | 1975-2010 | USA | General | Varying | P | FM/DM | Varying | [3] |
| CISNET E / MISCAN-Fadia | 2018 | E | 1975-2010 | USA | General | Varying | P/O | FM/DM | Varying | [4] |
|  | 2006 | D | 1975-2000 | USA | General | 30-79 | P | FM | Varying | [5] |
| CISNET GE / Spectrum | 2018 | E | 1972-2010 | USA | General | Varying | P | FM/DM/US | Varying | [6] |
|  | 2006 | D | 1890-1995 | USA | General | Varying | P | FM | Varying | [7] |
|  | 2005 | O | 1975-1979,  1995-2001 | USA | General | Varying | P | FM | Biennial | [8] |
|  | 2004 | O | 1975-1979,  1995-2001 | USA | Race-specific | 40+ | P | FM | Biennial | [9] |
| CISNET M | 2018 | O | 1975-2010 | USA | General | Varying | P | FM/DM | Varying | [10] |
| CISNET W/ UWBCS | 2018 | E | 1975-2010 | USA | General | 30-79 | P | FM/DM | Varying | [11] |
|  | 2013 | E | 1975-2000 | USA | Race-specific | Varying | P | FM | Varying | [12] |
|  | 2006 | D | 1975-2000 | USA | General | Varying | P | FM | Varying | [13] |
| Comas | 2014 | O | 2008 | Spain | General | 50-69 | P | FM/DM | Biennial | [14] |
| DES SD | 2015 | O | 2001-2010 | USA | General | 65+ | P | FM/DM | Annual | [15] |
| Forastero | 2010 | O | 2007 | Spain | General | Varying | P | FM | Varying | [16] |
| Gocgun | 2015 | O | 2012 | Canada | General | Varying | P | FM/DM | Varying | [17] |
| Gray | 2017 | O | 2013-2015 | UK | General | 50-70 | P | DM/US/MRI | Triennial | [18] |
| Gunsoy | 2014 | E | 1935-1940, 1971-2010 | UK | General | Varying | P | FM/DM | Annual/Triennial | [19] |
|  | 2012 | O | 1991-2010 | UK | General | 40-49 | P | FM/DM | Annual | [20] |
| Huang | 2020 | O | 1999-2010 | China | General | 30-80 | O | DM | Varying | [21] |
| Hunter | 2004 | O | 1973-1997, 2001 | Canada | General | 40-49 | P | FM | Biennial | [22] |
| LMIC | 2018 | O | 2012 | SEA, SSA | General | - | P | - | - | [23] |
| MDP | 2020 | O | 2012 | Peru, USA | General | Varying | P | FM | Varying | [24] |
| MISCAN | 2016 | E | 1975-2010 | NL | General | 50-75 | P | FM/DM | Biennial | [25] |
|  | 2011 | E | 2004-2006 | NL | General | 49-74 | P | FM/DM | Biennial | [26] |
|  | 2004 | E | 1975-1997 | NL | General | 50-69 | P | FM | Biennial | [27] |
|  | 1990 | O | 1977-1982 | NL | General | Varying | P | FM | Varying | [28] |
| OncoSim-Breast | 2022 | O | 1969-2013 | Canada | General | - | P | FM/DM | - | [29] |
| Ozanne | 2011 | O | 1978-2003 | USA | General | 50-74 | P | FM | Varying | [30] |
| POMDP | 2012 | O | 1975-2010 | USA | General/HR | Varying | P | FM/DM/CBE | Varying | [31] |
| Rafia | 2016 | O | 1988-1994, 2006 | UK | General | Varying | P | FM | Triennial | [32] |
| Rojnik | 2008 | O | 1980-1990, 1999-2001 | Slovenia | General | Varying | O | FM | Varying | [33] |
| Ryser | 2016 | O | 1991-2011 | USA | General | Varying | P | FM/DM/MRI | Varying | [34] |
| Schiller-Fruehwirth | 2017 | O | 1987-2011 | Austria | General | 45-69 | P/O | DM/US/MRI | Biennial | [35] |
| Schousboe | 2013 | O | 1975-2005 | USA | General | Varying | P | FM | Varying | [36] |
| Seigneurin | 2011 | O | 1991-2006 | France | General | 50-69 | P/O | FM | Biennial | [37] |
| Shih | 2021 | E | 1973-1979 | USA | General / DB | Varying | P | FM | Varying | [38] |
| Souza | 2013 | O | 2009-2011 | Brazil | General | 40-49 | P | FM/DM | Varying | [39] |
| Sun | 2018 | E | 2012-2013 | China | HR | 40-69 | O | DM/US | Varying | [40] |
| Tan | 2013 | O | 1978-1984 | Sweden | General | 40-74 | P | FM | Varying | [41] |
| Weedon-Fekjaer | 2020 | O | 1995-2002 | Norway | General | 50-69 | P/O | FM/DM | Varying | [42] |
| Wong | 2012 | E | 2002,2008 | HK | General | Varying | P | FM | Biennial | [43] |
|  | 2007 | O | 1983-1998 | HK | General | Varying | P | FM | Biennial | [44] |
| Yang | 2018 | O | 2011-2012 | China | General | 35-69 | O | DM/US/CBE | Varying | [45] |
| Yen | 2003 | O | - | Varying | General | Varying | P | FM | Varying | [46] |

^a^ Specific type of article: original (O), extension (E), description (D).

^b^ Country: United Kingdom (UK), United States (USA), Southeast Asia (SEA), Sub-Saharan Africa (SSA), the Netherlands (NL), Hong Kong (HK).

^c^ Population risk group: dense breasts (DB), ER-status (ER), High risk (HR).

^d^ Screening type: population-based (P), opportunistic (O).

^e^ Screening modality: digital mammography (DM), film mammography (FM), ultrasonography (US), magnetic resonance imaging (MRI), clinical breast examination (CBE).

## Appendix B.2.1. – Model overview – General characteristics – B) Applications

| Model | Publication Year | Study type^a^ | Years of input data | Country^b^ | Population  risk group^c^ | Screening age | Screening type^d^ | Screening modality^e^ | Screening  interval | Reference |
| --- | --- | --- | --- | --- | --- | --- | --- | --- | --- | --- |
| CISNET D | 2020 | A | 1975-2015 | USA | General | 30-79 | P | FM/DM | Varying | [47] |
|  | 2018 | A | 1975-2010 | USA | General | 62 | P | DM | - | [48] |
|  | 2018 | A | 1990-1997 | UK | General | 40-49 | P | FM | Annual | [49] |
|  | 2018 | A | 1975-2012 | USA | General | 30-79 | P | FM/DM | Varying | [50] |
|  | 2016 | A | 1975-2000 | USA | General | Varying | P | FM/DM | Varying | [51] |
|  | 2014 | A | 1975-2000 | USA | General (ER) | 30-79 | P | FM | Varying | [52] |
|  | 2014 | A | 1960-2000 | USA | General (DB) | Varying | P | FM/DM | Varying | [53] |
| CISNET E / MISCAN-Fadia | 2020 | A | 1975-2015 | USA | General | 30-79 | P | FM/DM | Varying | [47] |
|  | 2018 | A | 1975-2010 | USA | General | 62 | P | DM | - | [48] |
|  | 2018 | A | 1990-1997 | UK | General | 40-49 | P | FM | Annual | [49] |
|  | 2018 | A | 1975-2012 | USA | General | 30-79 | P | FM/DM | Varying | [50] |
|  | 2017 | A | 1975-2010 | USA | General | Varying | P | DM | Varying | [54] |
|  | 2016 | A | 1975-2000 | USA | General | Varying | P | FM/DM | Varying | [51] |
|  | 2016 | A | 2003-2011 | USA | General | 40-74 | P | DM | Varying | [55] |
|  | 2015 | A | 1975-2010 | USA | General | 40-64 | P | FM/DM | Varying | [56] |
|  | 2015 | A | 1975-2000 | USA | General | 50-74 | P | FM/DM/US | Biennial | [57] |
|  | 2015 | A | 2010 | USA | General | Varying | P | FM/DM | Biennial | [58] |
|  | 2014 | A | 1960-2000 | USA | General (DB) | Varying | P | FM/DM | Varying | [53] |
|  | 2013 | A | 1975-2000 | USA | General | Varying | P | FM/DM | Varying | [59] |
|  | 2012 | A | 1975-2000 | USA | General | Varying | P | FM/DM | Annual/Biennial | [60] |
|  | 2012 | A | 1970-2010 | USA | Race-specific | Varying | P | FM/DM | Varying | [61] |
|  | 2012 | A | 1975-2000 | USA | General | Varying | P | FM/DM | Annual/Biennial | [62] |
|  | 2011 | A | 1975-2010 | USA | Race-specific | 50-74 | P | FM/DM | Varying | [63] |
|  | 2011 | A | 1992 | UK | General | 50-62 | P | FM | Annual/Triennial | [64] |
|  | 2005 | A | 1975-2000 | USA | General | Varying | P | FM/DM | Varying | [65] |
| CISNET GE / Spectrum | 2018 | A | 1975-2010 | USA | General | 62 | P | DM | - | [48] |
|  | 2018 | A | 1975-2012 | USA | General | 30-79 | P | FM/DM | Varying | [50] |
|  | 2017 | A | 1975-2010 | USA | General | Varying | P | DM | Varying | [54] |
|  | 2016 | A | 1975-2000 | USA | General | Varying | P | FM/DM | Varying | [51] |
|  | 2015 | A | 1975-2010 | USA | General | 40-64 | P | FM/DM | Varying | [56] |
|  | 2015 | A | 1975-2000 | USA | General | 50-74 | P | FM/DM/US | Biennial | [57] |
|  | 2015 | A | 2010 | USA | General | Varying | P | FM/DM | Biennial | [58] |
|  | 2014 | A | 1960-2000 | USA | General (DB) | Varying | P | FM/DM | Varying | [53] |
|  | 2013 | A | 1975-2000 | USA | General | Varying | P | FM/DM | Varying | [59] |
|  | 2012 | A | 1975-2000 | USA | General | Varying | P | FM/DM | Annual/Biennial | [60] |
|  | 2012 | A | 1970-2010 | USA | Race-specific | Varying | P | FM/DM | Varying | [61] |
|  | 2012 | A | 1975-2000 | USA | General | Varying | P | FM/DM | Annual/Biennial | [62] |
|  | 2011 | A | 1975-2010 | USA | Race-specific | 50-74 | P | FM/DM | Varying | [63] |
|  | 2005 | A | 1975-2000 | USA | General | Varying | P | FM/DM | Varying | [65] |
| CISNET M | 2016 | A | 1975-2000 | USA | General | Varying | P | FM/DM | Varying | [51] |
|  | 2014 | A | 1960-2000 | USA | General (DB) | Varying | P | FM/DM | Varying | [53] |
|  | 2012 | A | 1975-2000 | USA | General | Varying | P | FM/DM | Annual/Biennial | [62] |
| CISNET W/ UWBCS | 2018 | A | 1975-2010 | USA | General | 62 | P | DM | - | [48] |
|  | 2018 | A | 1990-1997 | UK | General | 40-49 | P | FM | Annual | [49] |
|  | 2018 | A | 1975-2012 | USA | General | 30-79 | P | FM/DM | Varying | [50] |
|  | 2018 | A | 1975-2010 | USA | General | Varying | P | FM/DM | Varying | [66] |
|  | 2017 | A | 1975-2010 | USA | General | Varying | P | DM | Varying | [54] |
|  | 2016 | A | 1975-2000 | USA | General | Varying | P | FM/DM | Varying | [51] |
|  | 2015 | A | 1975-2000 | USA | General (DB) | 50-74 | P | DM/TS | Biennial | [67] |
|  | 2015 | A | 1975-2000 | USA | General | 50-74 | P | FM/DM/US | Biennial | [57] |
|  | 2014 | A | 1960-2000 | USA | General (DB) | Varying | P | FM/DM | Varying | [53] |
|  | 2012 | A | 1975-2000 | USA | General | Varying | P | FM/DM | Annual/Biennial | [60] |
|  | 2012 | A | 1975-2000 | USA | General | Varying | P | FM/DM | Annual/Biennial | [62] |
|  | 2008 | A | 1975-2000 | USA | General | 40+ | P | FM/DM | Varying | [68] |
|  | 2005 | A | 1975-2000 | USA | General | Varying | P | FM/DM | Varying | [65] |
| LMIC | 2018 | A | 2012 | SEA, SSA | General | 50-69 | P | DM | Biennial | [69] |
| MISCAN | 2012 | A | 1999-2006,  1997-2008,  1997-2005 | NL,  Canada  UK | HR | 25-70  25-65  35-49 | P | DM/MRI/US/CBE | Annual/6 months | [70] |
|  | 2008 | A | 1974-2005 | Switzerland | General | 50-69 | P/O | FM | Biennial | [71] |
|  | 2008 | A | 2000 | India | General | Varying | P | FM/CBE | Varying | [72] |
|  | 2006 | A | 2002 | NL | General | 50-74 | P | FM | Biennial | [73] |
|  | 1999 | A | - | NL | General | Varying | P | FM | Varying | [74] |
|  | 1999 | A | - | NL | General | Varying | P | FM | Varying | [75] |
|  | 1998 | A | 1989 | NL | General | Varying | P | FM | Varying | [76] |
|  | 1998 | A | 1985-1989 | Spain | General | 50-64 | P | FM | Biennial | [77] |
|  | 1998 | A | 1987 | UK | General | Varying | P | FM | Biennial/Triennial | [78] |
|  | 1997 | A | 1989-1990 | Germany | General | 50-69 | P | FM | Biennial | [79] |
|  | 1995 | A | 1989 | Sweden | General | Varying | P | FM | Varying | [80] |
| OncoSim-Breast | 2021 | A | 1920-2015 | Canada | General/HR | 40-74/50-74 | P | DM | Annual/Biennial | [81] |
| Shih | 2019 | A | 1973-1979, 1999-2012 | USA | General | Varying | P | FM/DM | Annual/Biennial | [82] |
| Wong | 2010 | A | - | HK | General | Varying | P | FM | Biennial | [83] |

^a^ Specific type of article: application (A).

^b^ Country: United Kingdom (UK), United States (USA), Southeast Asia (SEA), Sub-Saharan Africa (SSA), the Netherlands (NL), Hong Kong (HK).

^c^ Population risk group: dense breasts (DB), ER-status (ER), High risk (HR).

^d^ Screening type: population-based (P), opportunistic (O).

^e^ Screening modality: digital mammography (DM), film mammography (FM), ultrasonography (US), magnetic resonance imaging (MRI), clinical breast examination (CBE), tomosynthesis (TS).

## Appendix B.3. Assessment results

Appendix B.3.1. – Risk of bias


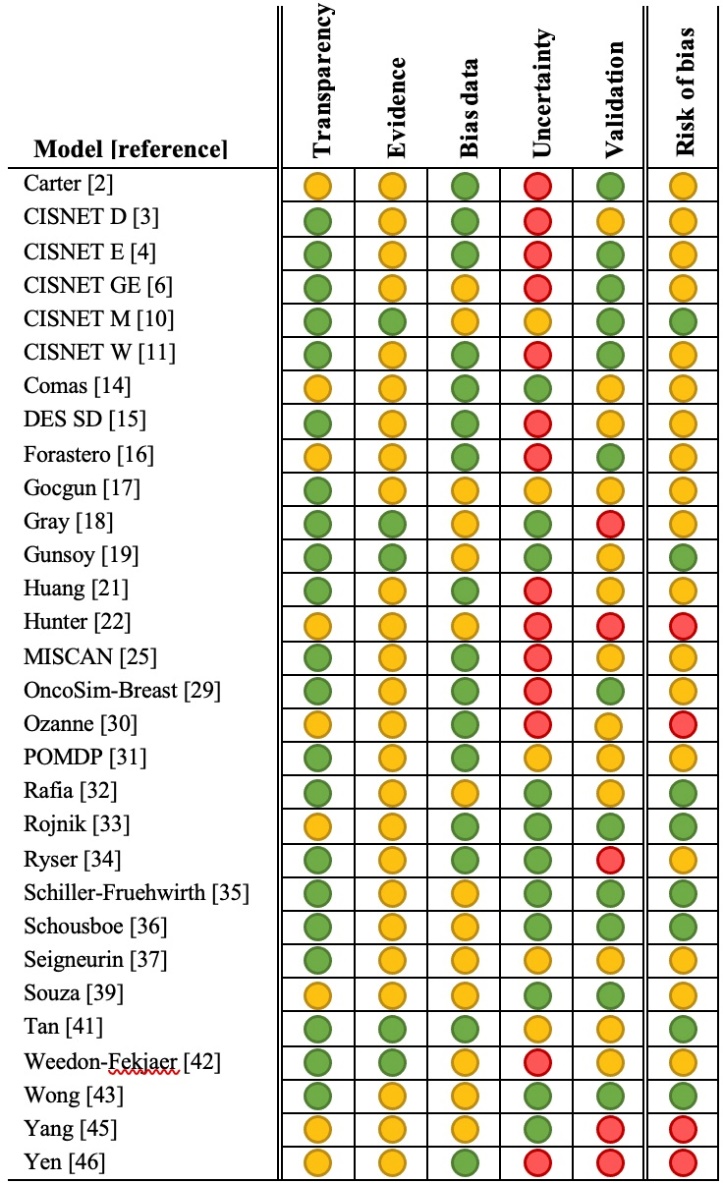


Appendix B.3.2. – Quality


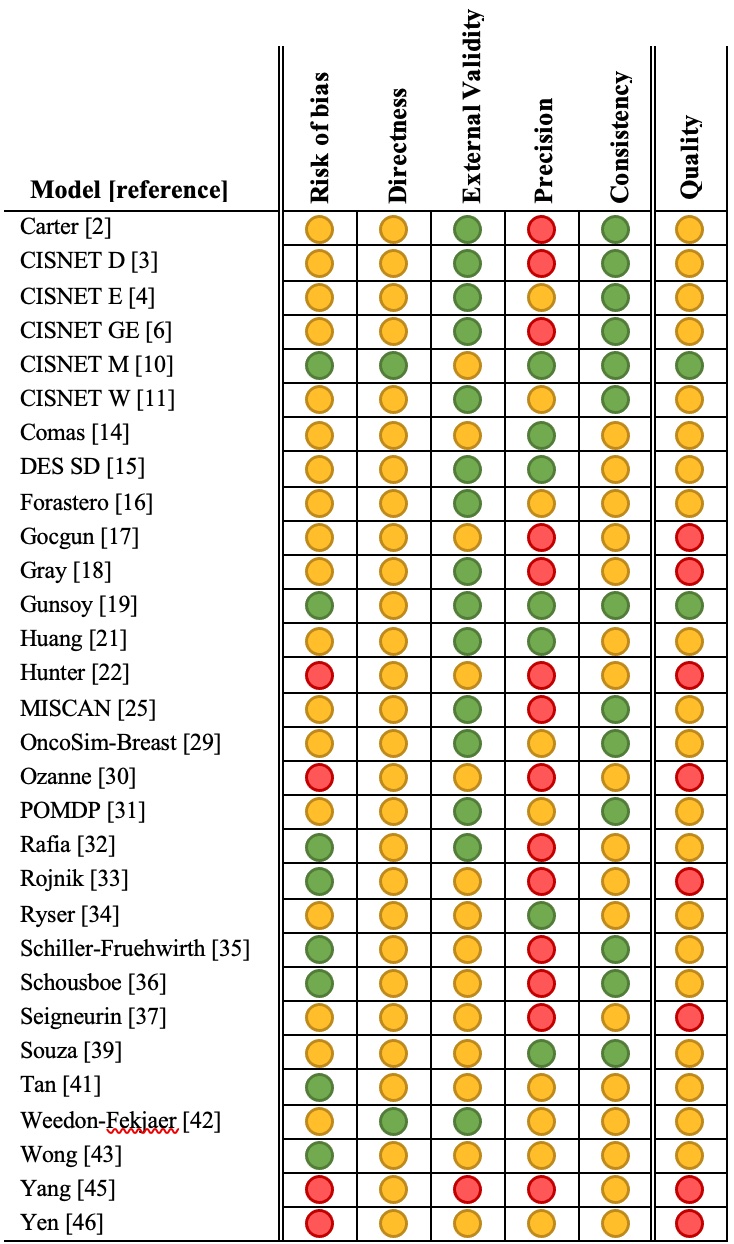


## References

[1] Carter JL, Coletti RJ, Harris RP., ‘Quantifying and monitoring overdiagnosis in cancer screening: a systematic review of methods’, *BMJ*, vol. 350:g7773, 2015, doi: 10.1136/bmj.g7773.

[2] Carter KJ, Castro F, Kessler E, Erickson B., ‘A computer model for the study of breast cancer’, *Comput Biol Med*, vol. 33, no. 4, pp. 345–360, 2003, doi: 10.1016/s0010-4825(03)00003-9.

[3] Lee SJ, Li X, Huang H, Zelen M., ‘The Dana-Farber CISNET Model for Breast Cancer Screening Strategies: An Update’, *Med Decis Making*, vol. 38, pp. 44S-53S, 2018, doi: 10.1177/0272989X17741634.

[4] van den Broek JJ, van Ravesteyn NT, Heijnsdijk EA, de Koning HJ., ‘Simulating the Impact of Risk-Based Screening and Treatment on Breast Cancer Outcomes with MISCAN-Fadia’, *Med Decis Making*, vol. 38, pp. 54S-65S, 2018, doi: 10.1177/0272989X17711928.

[5] Tan SY, van Oortmarssen GJ, de Koning HJ, Boer R, Habbema JD., ‘The MISCAN-Fadia continuous tumor growth model for breast cancer’, *J Natl Cancer Inst Monogr*, no. 36, pp. 56–65, 2006, doi: 10.1093/jncimonographs/lgj009.

[6] Schechter CB, Near AM, Jayasekera J, Chandler Y, Mandelblatt JS., ‘Structure, Function, and Applications of the Georgetown-Einstein (GE) Breast Cancer Simulation Model’, *Med Decis Making*, vol. 38, pp. 66S-77S, 2018, doi: 10.1177/0272989X17698685.

[7] Mandelblatt J, Schechter CB, Lawrence W, Yi B, Cullen J., ‘The SPECTRUM population model of the impact of screening and treatment on U.S. breast cancer trends from 1975 to 2000: principles and practice of the model methods’, *J Natl Cancer Inst Monogr*, pp. 47–55, 2006, doi: 10.1093/jncimonographs/lgj008.

[8] Mandelblatt JS, Schechter CB, Yabroff KR, Lawrence W, Dignam J, Extermann M, Fox S, Orosz G, Silliman R, Cullen J, Balducci L, ‘Breast Cancer in Older Women Research Consortium. Toward optimal screening strategies for older women. Costs, benefits, and harms of breast cancer screening by age, biology, and health status’, *J Gen Intern Med*, vol. 20, no. 6, pp. 487–496, 2005, doi: 10.1111/j.1525-1497.2005.0116.x.

[9] Mandelblatt JS, Schechter CB, Yabroff KR, Lawrence W, Dignam J, Muennig P, Chavez Y, Cullen J, Fahs M., ‘Benefits and costs of interventions to improve breast cancer outcomes in African American women’, *J Clin Oncol*, vol. 22, no. 13, pp. 2554–2566, 2004, doi: 10.1200/JCO.2004.05.009.

[10] Huang X, Li Y, Song J, Berry DA., ‘A Bayesian Simulation Model for Breast Cancer Screening, Incidence, Treatment, and Mortality’, *Med Decis Making*, vol. 38, pp. 78S-88S, 2018, doi: 10.1177/0272989X17714473.

[11] Alagoz O, Ergun MA, Cevik M, Sprague BL, Fryback DG, Gangnon RE, Hampton JM, Stout NK, Trentham-Dietz A., ‘The University of Wisconsin Breast Cancer Epidemiology Simulation Model: An Update’, *Med Decis Making*, vol. 38, pp. 99S-111S, 2018, doi: 10.1177/0272989X17711927.

[12] Batina NG, Trentham-Dietz A, Gangnon RE, Sprague BL, Rosenberg MA, Stout NK, Fryback DG, Alagoz O., ‘Variation in tumor natural history contributes to racial disparities in breast cancer stage at diagnosis’, *Breast Cancer Res Treat*, vol. 138, no. 2, pp. 519–528, 2013, doi: 10.1007/s10549-013-2435-z.

[13] Fryback DG, Stout NK, Rosenberg MA, Trentham-Dietz A, Kuruchittham V, Remington PL., ‘The Wisconsin Breast Cancer Epidemiology Simulation Model’, *J Natl Cancer Inst Monogr*, no. 36, pp. 37–47, 2006, doi: 10.1093/jncimonographs/lgj007.

[14] Comas M, Arrospide A, Mar J, Sala M, Vilaprinyó E, Hernández C, Cots F, Martínez J, Castells X., ‘Budget impact analysis of switching to digital mammography in a population-based breast cancer screening program: a discrete event simulation model’, *PLoS One*, vol. 9, no. 5, p. e97459, 2014, doi: 10.1371/journal.pone.0097459.

[15] Tejada JJ, Ivy JS, Wilson JR, Ballan MJ, et al., ‘Combined DES/SD model of breast cancer screening for older women, I: Natural-history simulation’, *IEE Transactions*, no. 47, pp. 600–619, 2015, doi: 10.1080/0740817X.2014.959671.

[16] Forastero C, Zamora LI, Guirado D, Lallena AM., ‘A Monte Carlo tool to simulate breast cancer screening programmes’, *Phys Med Biol*, vol. 55, no. 17, pp. 5213–5229, 2010, doi: 10.1088/0031-9155/55/17/021.

[17] Gocgun Y, Banjevic D, Taghipour S, Montgomery N, Harvey BJ, Jardine AK, Miller AB., ‘Cost-effectiveness of breast cancer screening policies using simulation’, *Breast*, vol. 24, no. 4, pp. 440–448, 2015, doi: 10.1016/j.breast.2015.03.012.

[18] Gray E, Donten A, Karssemeijer N, van Gils C, Evans DG, Astley S, Payne K., ‘Evaluation of a Stratified National Breast Screening Program in the United Kingdom: An Early Model-Based Cost-Effectiveness Analysis’, *Value Health*, vol. 20, no. 8, pp. 1100–1109, 2017, doi: 10.1016/j.jval.2017.04.012.

[19] Gunsoy NB, Garcia-Closas M, Moss SM., ‘Estimating breast cancer mortality reduction and overdiagnosis due to screening for different strategies in the United Kingdom’, *Br J Cancer*, vol. 110, no. 10, pp. 2412–2419, 2014, doi: 10.1038/bjc.2014.206.

[20] Gunsoy NB, Garcia-Closas M, Moss SM., ‘Modelling the overdiagnosis of breast cancer due to mammography screening in women aged 40 to 49 in the United Kingdom’, *Breast Cancer Res*, vol. 14, no. 6, p. R152, 2012, doi: 10.1186/bcr3365.

[21] Huang Y, Li Q, Torres-Rueda S, Li J., ‘The Structure and Parameterization of the Breast Cancer Transition Model Among Chinese Women’, *Value Health Reg Issues*, vol. 21, pp. 29–38, 2020, doi: 10.1016/j.vhri.2019.05.003.

[22] Hunter DJ, Drake SM, Shortt SE, Dorland JL, Tran N., ‘Simulation modeling of change to breast cancer detection age eligibility recommendations in Ontario, 2002-2021’, *Cancer Detect Prev*, vol. 28, no. 6, pp. 453–460, 2004, doi: 10.1016/j.cdp.2004.08.003.

[23] Gopalappa C, Guo J, Meckoni P, Munkhbat B, Pretorius C, Lauer J, Ilbawi A, Bertram M., ‘A Two-Step Markov Processes Approach for Parameterization of Cancer State-Transition Models for Low- and Middle-Income Countries’, *Med Decis Making*, vol. 38, no. 4, pp. 520–530, 2018, doi: 10.1177/0272989X18759482.

[24] Bansal S, Deshpande V, Zhao X, Lauer JA, Meheus F, Ilbawi A, Gopalappa C., ‘Analysis of Mammography Screening Schedules under Varying Resource Constraints for Planning Breast Cancer Control Programs in Low- and Middle-Income Countries: A Mathematical Study’, *Med Decis Making*, vol. 40, no. 3, pp. 364–378, 2020, doi: 10.1177/0272989X20910724.

[25] van Luijt PA, Heijnsdijk EA, Fracheboud J, Overbeek LI, Broeders MJ, Wesseling J, den Heeten GJ, de Koning HJ., ‘The distribution of ductal carcinoma in situ (DCIS) grade in 4232 women and its impact on overdiagnosis in breast cancer screening’, *Breast Cancer Res*, vol. 18, no. 1, p. 47, 2016, doi: 10.1186/s13058-016-0705-5.

[26] de Gelder R, Fracheboud J, Heijnsdijk EA, den Heeten G, Verbeek AL, Broeders MJ, Draisma G, de Koning HJ., ‘Digital mammography screening: weighing reduced mortality against increased overdiagnosis’, *Prev Med*, vol. 53, no. 3, pp. 134–140, 2011, doi: 10.1016/j.ypmed.2011.06.009.

[27] Vervoort MM, Draisma G, Fracheboud J, van de Poll-Franse LV, de Koning HJ., ‘Trends in the usage of adjuvant systemic therapy for breast cancer in the Netherlands and its effect on mortality’, *Br J Cancer*, vol. 91, no. 2, pp. 242–247, 2004, doi: 10.1038/sj.bjc.6601969.

[28] van Oortmarssen GJ, Habbema JD, van der Maas PJ, de Koning HJ, et al., ‘A model for breast cancer screening’, *Cancer*, vol. 66, no. 7, pp. 1601–12, 1990, doi: 10.1002/1097-0142(19901001)66:7<1601::aid-cncr2820660727>3.0.co;2-o.

[29] Yong JHE, Nadeau C, Flanagan WM, Coldman AJ, Asakawa K, Garner R, Fitzgerald N, Yaffe MJ, Miller AB., ‘The OncoSim-Breast Cancer Microsimulation Model’, *Curr. Oncol.*, vol. 29, pp. 1619–1633, 2022, doi: https://doi.org/ 10.3390/curroncol29030136.

[30] Ozanne EM, Shieh Y, Barnes J, Bouzan C, Hwang ES, Esserman LJ., ‘Characterizing the impact of 25 years of DCIS treatment’, *Breast Cancer Res Treat*, vol. 129, no. 1, pp. 165–173, 2011, doi: 10.1007/s10549-011-1430-5.

[31] Ayer T, Alagoz O, Stout NK., ‘A POMDP Approach to Personalize Mammography Screening Decisions’, *Operations Research*, vol. 60, no. 5, pp. 1019–1034, 2012, doi: http://dx.doi.org/10.1287/opre. 1110.1.

[32] Rafia R, Brennan A, Madan J, Collins K, Reed MW, Lawrence G, Robinson T, Greenberg D, Wyld L., ‘Modeling the Cost-Effectiveness of Alternative Upper Age Limits for Breast Cancer Screening in England and Wales’, *Value Health*, vol. 19, no. 4, pp. 404–412, 2016, doi: 10.1016/j.jval.2015.06.006.

[33] Rojnik K, Naversnik K, Mateovic-Rojnik T, et al., ‘Probabilistic Cost-Effectiveness Modeling of Different Breast Cancer Screening Policies in Slovenia’, *Value in Health*, vol. 11, no. 2, pp. 139–148, 2008, doi: https://doi.org/10.1111/j.1524-4733.2007.00223.x.

[34] Ryser MD, Worni M, Turner EL, Marks JR, Durrett R, Hwang ES., ‘Outcomes of Active Surveillance for Ductal Carcinoma in Situ: A Computational Risk Analysis’, *J Natl Cancer Inst*, vol. 108, no. 5, 2015, doi: 10.1093/jnci/djv372.

[35] Schiller-Fruehwirth I, Jahn B, Einzinger P, Zauner G, Urach C, Siebert U., ‘The Long-Term Effectiveness and Cost Effectiveness of Organized versus Opportunistic Screening for Breast Cancer in Austria’, *Value Health*, vol. 20, no. 8, pp. 1048–1057, 2017, doi: 10.1016/j.jval.2017.04.009.

[36] Schousboe JT, Kerlikowske K, Loh A, Cummings SR, ‘Personalizing mammography by breast density and other risk factors for breast cancer: analysis of health benefits and cost-effectiveness’, *Ann Intern Med*, vol. 155, 2011, doi: 10.7326/0003-4819-155-1-201107050-00003.

[37] Seigneurin A, Francois O, Labarere J, et al., ‘Overdiagnosis from non-progressive cancer detected by screening mammography: stochastic simulation study with calibration to population based registry data’, *BMJ*, vol. 343, 2011, doi: https://doi.org/10.1136/bmj.d7017.

[38] Shih YT, Dong W, Xu Y, Etzioni R, Shen Y., ‘Incorporating Baseline Breast Density When Screening Women at Average Risk for Breast Cancer: A Cost-Effectiveness Analysis’, *Ann Intern Med.*, vol. 174, no. 5, pp. 602–612, 2021, doi: 10.7326/M20-2912.

[39] Souza FH, Polanczyk CA., ‘Is Age-targeted full-field digital mammography screening cost-effective in emerging countries? A micro simulation model’, *Springerplus*, vol. 2, p. 366, 2013, doi: 10.1186/2193-1801-2-366.

[40] Sun L, Legood R, Sadique Z, Dos-Santos-Silva I, Yang L., ‘Cost-effectiveness of risk-based breast cancer screening programme, China’, *Bull World Health Organ*, vol. 96, no. 8, pp. 568–577, 2018, doi: 10.2471/BLT.18.207944.

[41] Tan KH, Simonella L, Wee HL, Roellin A, Lim YW, Lim WY, Chia KS, Hartman M, Cook AR., ‘Quantifying the natural history of breast cancer’, *Br J Cancer*, vol. 109, no. 8, pp. 2035–2043, 2013, doi: 10.1038/bjc.2013.471.

[42] Weedon-Fekjær H, Li X, Lee S., ‘Estimating the natural progression of non-invasive ductal carcinoma in situ breast cancer lesions using screening data’, *J Med Screen*, vol. 28, no. 3, pp. 302–310, 2021, doi: 10.1177/0969141320945736.

[43] Wong IO, Tsang JW, Cowling BJ, Leung GM., ‘Optimizing resource allocation for breast cancer prevention and care among Hong Kong Chinese women’, *Cancer*, vol. 118, no. 18, pp. 4394–4403, 2012, doi: 10.1002/cncr.27448.

[44] Wong IO, Kuntz KM, Cowling BJ, Lam CL, Leung GM, ‘Cost effectiveness of mammography screening for Chinese women’, *Cancer*, vol. 110, no. 4, pp. 885–895, 2007, doi: 10.1002/cncr.22848.

[45] Yang L, Wang J, Cheng J, Wang Y, Lu W., ‘Quality assurance target for community-based breast cancer screening in China: a model simulation’, *BMC Cancer*, vol. 18, no. 1, p. 261, 2018, doi: 10.1186/s12885-018-4168-1.

[46] Yen MF, Tabár L, Vitak B, Smith RA, Chen HH, Duffy SW., ‘Quantifying the potential problem of overdiagnosis of ductal carcinoma in situ in breast cancer screening’, *Eur J Cancer*, vol. 39, no. 12, pp. 1746–1754, 2003, doi: 10.1016/s0959-8049(03)00260-0.

[47] Chootipongchaivat S, van Ravesteyn NT, Li X, Huang H, Weedon-Fekjær H, Ryser MD, Weaver DL, Burnside ES, Heckman-Stoddard BM, de Koning HJ, Lee SJ., ‘Modeling the natural history of ductal carcinoma in situ based on population data’, *Breast Cancer Res*, vol. 22, no. 1, p. 53, 2020, doi: 10.1186/s13058-020-01287-6.

[48] van den Broek JJ, van Ravesteyn NT, Mandelblatt JS, Cevik M, Schechter CB, Lee SJ, Huang H, Li Y, Munoz DF, Plevritis SK, de Koning HJ, Stout NK, van Ballegooijen M., ‘Comparing CISNET Breast Cancer Models Using the Maximum Clinical Incidence Reduction Methodology’, *Med Decis Making*, vol. 38, pp. 112S-125S, 2018, doi: 10.1177/0272989X17743244.

[49] van den Broek JJ, van Ravesteyn NT, Mandelblatt JS, Huang H, Ergun MA, Burnside ES, Xu C, Li Y, Alagoz O, Lee SJ, Stout NK, Song J, Trentham-Dietz A, Plevritis SK, Moss SM, de Koning HJ., ‘Comparing CISNET Breast Cancer Incidence and Mortality Predictions to Observed Clinical Trial Results of Mammography Screening from Ages 40 to 49’, *Med Decis Making*, vol. 38, pp. 140S-150S, 2018, doi: 10.1177/0272989X17718168.

[50] Plevritis SK, Munoz D, Kurian AW, Stout NK, Alagoz O, Near AM, Lee SJ, van den Broek JJ, Huang X, et al., ‘Association of Screening and Treatment With Breast Cancer Mortality by Molecular Subtype in US Women, 2000-2012’, *JAMA*, vol. 319, no. 2, pp. 154–164, 2018, doi: 10.1001/jama.2017.19130.

[51] Mandelblatt JS, Stout NK, Schechter CB, van den Broek JJ, et al., ‘Collaborative Modeling of the Benefits and Harms Associated With Different U.S. Breast Cancer Screening Strategies’, *Ann Intern Med*, vol. 164, no. 4, pp. 215–25, 2016, doi: 10.7326/M15-1536.

[52] Munoz D, Near AM, van Ravesteyn NT, Lee SJ, Schechter CB, Alagoz O, Berry DA, Burnside ES, Chang Y, Chisholm G, de Koning HJ, Ali Ergun M, Heijnsdijk EA, Huang H, Stout NK, Sprague BL, Trentham-Dietz A, Mandelblatt JS, Plevritis SK., ‘Effects of screening and systemic adjuvant therapy on ER-specific US breast cancer mortality’, *J Natl Cancer Inst*, vol. 106, no. 11, 2014, doi: 10.1093/jnci/dju289.

[53] Stout NK, Lee SJ, Schechter CB, Kerlikowske K, Alagoz O, Berry D, Buist DS, Cevik M, Chisholm G, de Koning HJ, Huang H, Hubbard RA, Miglioretti DL, Munsell MF, Trentham-Dietz A, van Ravesteyn NT, Tosteson AN, Mandelblatt JS., ‘Benefits, harms, and costs for breast cancer screening after US implementation of digital mammography’, *J Natl Cancer Inst*, vol. 106, no. 6, 2014, doi: 10.1093/jnci/dju092.

[54] Trentham-Dietz A, Kerlikowske K, Stout NK, Miglioretti DL, et al., ‘Tailoring Breast Cancer Screening Intervals by Breast Density and Risk for Women Aged 50 Years or Older: Collaborative Modeling of Screening Outcomes’, *Ann Intern Med*, vol. 165, no. 10, pp. 700–712, 2016, doi: 10.7326/M16-0476.

[55] Miglioretti DL, Lange J, van den Broek JJ, Lee CI, et al., ‘Radiation-Induced Breast Cancer Incidence and Mortality From Digital Mammography Screening: A Modeling Study’, *Ann Intern Med*, vol. 164, no. 6, pp. 205–14, 2016, doi: 10.7326/M15-1241.

[56] van Ravesteyn NT, van Lier L, Schechter CB, Ekwueme DU, Royalty J, Miller JW, Near AM, Cronin KA, Heijnsdijk EA, Mandelblatt JS, de Koning HJ., ‘Transition from film to digital mammography: impact for breast cancer screening through the national breast and cervical cancer early detection program’, *Am J Prev Med*, vol. 48, no. 5, pp. 535–542, 2015, doi: 10.1016/j.amepre.2014.11.010.

[57] Sprague BL, Stout NK, Schechter C, van Ravesteyn NT, et al., ‘Benefits, harms, and cost-effectiveness of supplemental ultrasonography screening for women with dense breasts’, *Ann Intern Med*, vol. 162, no. 3, pp. 157–166, 2015, doi: 10.7326/M14-0692.

[58] Lansdorp-Vogelaar I, Gulati R, Mariotto AB, Schechter CB, et al., ‘Personalizing age of cancer screening cessation based on comorbid conditions: model estimates of harms and benefits’, *Ann Intern Med*, vol. 161, no. 2, pp. 104–112, 2014, doi: 10.7326/M13-2867.

[59] Mandelblatt J, van Ravesteyn N, Schechter C, Chang Y, et al., ‘Which strategies reduce breast cancer mortality most? Collaborative modeling of optimal screening, treatment, and obesity prevention’, *Cancer*, vol. 119, no. 14, pp. 2541–8, 2013, doi: 10.1002/cncr.28087.

[60] van Ravesteyn NT, Miglioretti DL, Stout NK, Lee SJ, et al., ‘Tipping the balance of benefits and harms to favor screening mammography starting at age 40 years: a comparative modeling study of risk’, *Ann Intern Med*, vol. 156, no. 9, pp. 609–617, 2012, doi: 10.7326/0003-4819-156-9-201205010-00002.

[61] Chang Y, Schechter CB, van Ravesteyn NT, Near AM, et al., ‘Collaborative modeling of the impact of obesity on race-specific breast cancer incidence and mortality’, *Breast Cancer Res Treat*, vol. 136, no. 3, pp. 823–35, 2012, doi: 10.1007/s10549-012-2274-3.

[62] Mandelblatt JS, Cronin KA, Bailey S, Berry DA, et al., ‘Effects of mammography screening under different screening schedules: model estimates of potential benefits and harms’, *Ann Intern Med*, vol. 151, no. 10, pp. 738–47, 2009, doi: 10.7326/0003-4819-151-10-200911170-00010.

[63] van Ravesteyn NT, Schechter CB, Near AM, Heijnsdijk EA, Stoto MA, Draisma G, de Koning HJ, Mandelblatt JS., ‘Race-specific impact of natural history, mammography screening, and adjuvant treatment on breast cancer mortality rates in the United States’, *Cancer Epidemiol Biomarkers Prev*, vol. 20, no. 1, pp. 112–122, 2011, doi: 10.1158/1055-9965.EPI-10-0944.

[64] van Ravesteyn NT, Heijnsdijk EA, Draisma G, de Koning HJ, ‘Prediction of higher mortality reduction for the UK Breast Screening Frequency Trial: a model-based approach on screening intervals’, *Br J Cancer*, vol. 105, no. 7, pp. 1082–88, 2011, doi: 10.1038/bjc.2011.300.

[65] Berry DA, Cronin KA, Plevritis SK, Fryback DG, et al., ‘Cancer Intervention and Surveillance Modeling Network (CISNET) Collaborators. Effect of screening and adjuvant therapy on mortality from breast cancer’, *N Engl J Med*, vol. 353, no. 17, pp. 1784–92, 2005, doi: 10.1056/NEJMoa050518.

[66] Trentham-Dietz A, Ergun MA, Alagoz O, Stout NK, Gangnon RE, Hampton JM, Dittus K, James TA, Vacek PM, Herschorn SD, Burnside ES, Tosteson ANA, Weaver DL, Sprague BL., ‘Comparative effectiveness of incorporating a hypothetical DCIS prognostic marker into breast cancer screening’, *Breast Cancer Res Treat*, vol. 168, no. 1, pp. 229–239, 2018, doi: 10.1007/s10549-017-4582-0.

[67] Lee CI, Cevik M, Alagoz O, Sprague BL, et al., ‘Comparative effectiveness of combined digital mammography and tomosynthesis screening for women with dense breasts’, *Radiology*, vol. 274, no. 3, pp. 772–780, 2015, doi: 10.1148/radiol.14141237.

[68] Tosteson AN, Stout NK, Fryback DG, Acharyya S, et al., ‘Cost-effectiveness of digital mammography breast cancer screening’, *Ann Intern Med*, vol. 148, no. 1, 2008, doi: 10.7326/0003-4819-148-1-200801010-00002.

[69] Ralaidovy AH, Gopalappa C, Ilbawi A, Pretorius C, Lauer JA., ‘Cost-effective interventions for breast cancer, cervical cancer, and colorectal cancer: new results from WHO-CHOICE’, *Cost Eff Resour Alloc*, vol. 16, p. 38, 2018, doi: 10.1186/s12962-018-0157-0.

[70] Heijnsdijk EA, Warner E, Gilbert FJ, Tilanus-Linthorst MM, Evans G, Causer PA, Eeles RA, Kaas R, Draisma G, Ramsay EA, Warren RM, Hill KA, Hoogerbrugge N, Wasser MN, Bergers E, Oosterwijk JC, Hooning MJ, Rutgers EJ, Klijn JG, et al., ‘Differences in natural history between breast cancers in BRCA1 and BRCA2 mutation carriers and effects of MRI screening-MRISC, MARIBS, and Canadian studies combined’, *Cancer Epidemiol Biomarkers Prev*, vol. 21, no. 9, pp. 1458–1468, 2012, doi: 10.1158/1055-9965.EPI-11-1196.

[71] de Gelder R, Bulliard JL, de Wolf C, Fracheboud J, et al., ‘Cost-effectiveness of opportunistic versus organised mammography screening in Switzerland’, *Eur J Cancer*, vol. 45, no. 1, pp. 127–138, 2009, doi: 10.1016/j.ejca.2008.09.015.

[72] Okonkwo QL, Draisma G, der Kinderen A, Brown ML, et al., ‘Breast cancer screening policies in developing countries: a cost-effectiveness analysis for India’, *J Natl Cancer Inst*, vol. 100, no. 18, pp. 1290–300, 2008, doi: 10.1093/jnci/djn292.

[73] de Koning HJ, Draisma G, Fracheboud J, de Bruijn A., ‘Overdiagnosis and overtreatment of breast cancer: microsimulation modelling estimates based on observed screen and clinical data’, *Breast Cancer Res*, vol. 8, no. 1, p. 202, 2006, doi: 10.1186/bcr1369.

[74] Boer R, de Koning H, van Oortmarssen G, Warmerdam P, van der Maas P., ‘Stage distribution at first and repeat examinations in breast cancer screening’, *J Med Screen*, vol. 6, no. 3, pp. 132–138, 1999, doi: 10.1136/jms.6.3.132.

[75] Boer R, de Koning HJ, van der Maas PJ., ‘A longer breast carcinoma screening interval for women age older than 65 years?’, *Cancer*, vol. 86, no. 8, pp. 1506–1510, 1999, doi: 10.1002/(sici)1097-0142(19991015)86:8<1506::aid-cncr17>3.0.co;2-2.

[76] Beemsterboer PM, Warmerdam PG, Boer R, de Koning HJ., ‘Radiation risk of mammography related to benefit in screening programmes: a favourable balance?’, *J Med Screen*, vol. 5, no. 2, pp. 81–87, 1998, doi: 10.1136/jms.5.2.81.

[77] Beemsterboer PMM, Warmerdam PG, Boer R, Borras JM, et al., ‘Screening for breast cancer in Catalonia: Which policy is to be preferred?’, *Eur J Public Health*, vol. 8, no. 3, pp. 241–246, 1998.

[78] Boer R, de Koning H, Threlfall A, Warmerdam P, et al., ‘Cost effectiveness of shortening screening interval or extending age range of NHS breast screening programme: computer simulation study’, *BMJ*, vol. 317, no. 7155, pp. 376–9, 1998, doi: 10.1136/bmj.317.7155.376.

[79] Warmerdam PG, de Koning HJ, Boer R, Beemsterboer PM, Dierks ML, Swart E, Robra BP., ‘Quantitative estimates of the impact of sensitivity and specificity in mammographic screening in Germany’, *J Epidemiol Community Health*, vol. 51, no. 2, pp. 180–186, 1997, doi: 10.1136/jech.51.2.180.

[80] de Koning HJ, Boer R, Warmerdam PG, Beemsterboer PM, van der Maas PJ., ‘Quantitative interpretation of age-specific mortality reductions from the Swedish breast cancer-screening trials’, *J Natl Cancer Inst*, vol. 87, no. 16, pp. 1217–1223, 1995, doi: 10.1093/jnci/87.16.1217.

[81] Yong JH, Mainprize JG, Yaffe MJ, Ruan Y, Poirier AE, Coldman A, Nadeau C, Iragorri N, Hilsden RJ, Brenner DR., ‘The impact of episodic screening interruption: COVID-19 and population-based cancer screening in Canada’, *J Med Screen*, vol. 28, no. 2, pp. 100–107, 2021, doi: 10.1177/0969141320974711.

[82] Tina Shih YC, Dong W, Xu Y, Shen Y., ‘Assessing the Cost-Effectiveness of Updated Breast Cancer Screening Guidelines for Average-Risk Women’, *Value Health*, vol. 22, no. 2, pp. 185–193, 2019, doi: 10.1016/j.jval.2018.07.880.

[83] Wong IO, Kuntz KM, Cowling BJ, Lam CL, Leung GM., ‘Cost-effectiveness analysis of mammography screening in Hong Kong Chinese using state-transition Markov modelling’, *Hong Kong Med J*, vol. 16, pp. 38–41, 2010.
